# Interleukin-15 and interferon-γ participate in the cross-talk between natural killer and monocytic cells required for tumour necrosis factor production

**DOI:** 10.1186/ar1955

**Published:** 2006-05-09

**Authors:** Isidoro González-Álvaro, Carmen Domínguez-Jiménez, Ana M Ortiz, Vanessa Núñez-González, Pedro Roda-Navarro, Elena Fernández-Ruiz, David Sancho, Francisco Sánchez-Madrid

**Affiliations:** 1 Servicio de Reumatologia, Hospital Universitario de la Princesa, c/ Diego de León 62, 28006 Madrid, Spain; 2 Unidad de Biología Molecular, Hospital Universitario de la Princesa, c/ Diego de León 62, 28006 Madrid, Spain; 3 Current address: Department of Pathology, University of Cambridge, Tennis Court Road, Cambridge, CB2 1QP, UK; 4 Servicio de Inmunologia, Hospital Universitario de la Princesa, c/ Diego de León 62, 28006 Madrid, Spain

## Abstract

We have characterized the lymphocyte subset and the receptor molecules involved in inducing the secretion of TNF by monocytic cells *in vitro*. The TNF secreted by monocytic cells was measured when they were co-cultured with either resting or IL-15-stimulated lymphocytes, T cells, B cells or natural killer (NK) cells isolated from the peripheral blood of healthy subjects and from the synovial fluid from patients with inflammatory arthropathies. Co-culture with IL-15-activated peripheral blood or synovial fluid lymphocytes induced TNF production by monocytic cells within 24 hours, an effect that was mainly mediated by NK cells. In turn, monocytic cells induced CD69 expression and IFN-γ production in NK cells, an effect that was mediated mainly by β_2 _integrins and membrane-bound IL-15. Furthermore, IFN-γ increased the production of membrane-bound IL-15 in monocytic cells. Blockade of β_2 _integrins and membrane-bound IL-15 inhibited TNF production, whereas TNF synthesis increased in the presence of anti-CD48 and anti-CD244 (2B4) monoclonal antibodies. All these findings suggest that the cross-talk between NK cells and monocytes results in the sustained stimulation of TNF production. This phenomenon might be important in the pathogenesis of conditions such as rheumatoid arthritis in which the synthesis of TNF is enhanced.

## Introduction

Rheumatoid arthritis (RA) is the most common chronic polyarthritis and the autoimmune foundation of its pathogenesis was established in the mid-twentieth century [1]. The importance of self-reactivity in RA was first suggested by the identification of rheumatoid factor, and attention subsequently became focused on T cells as the cornerstone in the aetiology and pathogenesis of this condition [1]. Memory T lymphocytes bearing different activation markers (CD69, CD71) form the most prominent subset of infiltrating cells in rheumatoid synovium [2,3]. In addition, the strong genetic-link between RA and class II MHC molecules suggests that CD4^+ ^T cells might be important in the development of the disease [1]. However, the low concentrations of T cell-derived cytokines such as IL-2, coupled with the absence of T cell proliferation and clonal expansion in the rheumatoid synovium, has attenuated the interest in CD4^+ ^T cells in RA [4]. Furthermore, the efficacy of anti-CD4 therapy in RA is far lower than that directed against TNF, IL-1 or CD20 [5-7].

Although it is clear that TNF is currently the most important cytokine in the pathogenesis of RA, the mechanisms involved in the perpetuation of TNF production in the rheumatoid synovium are not yet fully understood [1,8]. In this regard, it has been proposed that antigen-independent T lymphocyte activation might be involved in chronic TNF production in the rheumatoid synovium through cell–cell interactions [9-11]. In addition, it has been suggested that natural killer (NK) cells might also be involved in the intercellular contacts that induce TNF production in monocytes and dendritic cells [12-14]. To further understand the cellular and molecular interactions that regulate TNF production by monocytes/macrophages, we have studied the effect of different lymphocyte subsets in this process, as well as the involvement of functional relevant molecules.

## Materials and methods

### Antibodies and reagents

The mAbs TP1/55 (anti-CD69), HP2/6 (anti-CD4), Lia3/2 (anti-CD18), B942 (anti-CD8) and DR (anti-HLA-DR) have been described previously [15,16]. The mAbs T3b (anti-CD3) and BU12 (anti-CD19) were generously donated by Dr J De Vries (DNAX, Palo Alto, CA, USA). The BAB281 (anti-NKp46), MA152 (anti-NKp80), z199 (anti CD94/NKG2A) and KD1 (anti-CD16) mAbs were kindly provided by Dr A Moretta (Universita degli Studi di Genova, Genova, Italy). Phycoerythrin-conjugated Leu-19 (anti-CD56), Leu-19 (anti-CD56 pure) and isotype-matched controls were purchased from Becton Dickinson (Mountain View, CA, USA). Anti-human NKG2D (MAB139), blocking anti-human IL-15 (MAB647), anti-human CD244 (2B4; MAB1039) and the negative control MAB002 mAb were obtained from R&D Systems (Abingdon, Oxon., UK). The anti-human CD244 (2-69) was from BD-Pharmigen (San Diego, CA, USA) and the anti-human CD48 (156-4H9) was from NeoMarkers (Freemont, CA, USA).

Recombinant human IL-15, IFN-γ, TNF and IL-1 were supplied by PeproTech EC, Ltd (London, UK). FCS was purchased from Boehringer Mannheim (Mannheim, Germany), RPMI 1640 medium, Dulbecco's modified Eagle's medium, penicillin and streptomycin were provided by BioWhittaker (Verviers, Belgium) and L-glutamine by Gibco BRL (Paisley, Renfrewshire, Scotland). Lipopolysaccharide was supplied by Sigma Diagnostics (St Louis, MO, USA).

### Isolation of lymphocyte subsets

Peripheral blood lymphocytes (PBL) were isolated from healthy donors by Histopaque-1077 density-gradient centrifugation (Sigma Diagnostics). This was followed by the removal of monocytes by adhesion for 1 hour to Petri dishes (Costar, Cambridge, MA, USA) in RPMI 1640 medium supplemented with 10% FCS at 37°C. The lymphocyte-enriched fraction contained less than 1% CD14^+ ^cells. CD4^+ ^and CD8^+ ^cells were then obtained by negative selection with Subset Enrichment Column kits (R&D Systems). Cell purity was determined by flow cytometry and was always greater than 90% for CD4^+ ^and 95% for CD8^+ ^T lymphocytes. NK cells were purified by negative selection with goat anti-mouse IgG Dynabeads (Dynal Biotech, Oslo, Norway) previously coupled to anti-CD3, anti-CD4 and anti-HLA-DR mAbs. After a second round of selection with beads coupled to CD3 and CD19 mAbs (Dynal Biotech) the cell population obtained was more than 95% CD56^+ ^with less than 1% CD3^+ ^cells.

In other experiments, PBL were depleted of T cells, B cells or NK cells, and the NK-depleted PBL were obtained by incubating the PBL with immunomagnetic beads coupled to BAB281 (anti-NKp46), KD1 (anti-CD16) and Leu-19 (anti-CD56) mAbs. This process was repeated and the cell population obtained was less than 1% CD56^+^. B cell-depleted PBL and T cell-depleted PBL populations were isolated by using the same procedure with the anti-CD19 and anti-HLA-DR mAbs, yielding a B cell-depleted PBL population that was less than 0.5% CD19^+^. When anti-CD3, anti-CD4 and anti-CD8 was used, the T cell-depleted PBL population was less than 1% CD3^+^.

### Monocytic cells

Most experiments were performed with the human monocytic leukaemic cell line THP-1 obtained from ATCC/LGC Promochem (Barcelona, Spain). These cells were maintained in culture with RPMI 1640 medium supplemented with 10% heat-inactivated FCS, penicillin (100 U/ml) and streptomycin (100 μg/ml) at 37°C in a humidified atmosphere consisting of 5% CO_2 _.

In experiments performed with human peripheral blood monocytes, these cells were obtained with the following purification procedure: peripheral blood mononuclear cells were obtained by Histopaque-1077 density-gradient centrifugation and resuspended in RPMI 1640 medium supplemented with 10% FCS. A sample of this cellular suspension was analysed through a Hitachi Coulter counter to determine the concentration of monocytes. A volume containing 10^5 ^monocytes was then added to each well in 24-well plates (Costar) to allow the attachment of monocytes and, after 1 hour at 37°C, wells were washed three timed with RPMI 1640 medium. The population attached to wells was more than 90% CD14^+ ^after cell detachment and flow cytometry analysis. To perform co-culture assays, the autologous lymphocytes were treated as described above and co-cultured with the monocytes at a 10:1 ratio (10^6 ^lymphocytes or subpopulations per 10^5 ^monocytes attached at the wells of 24-well plates).

### Patients and synovial fluid samples

Synovial fluid samples were obtained, with previous oral informed consent, from patients attending our out-patient clinic. Diagnoses included RA (*n *= 5), seronegative spondyloarthropathies (*n *= 6) and crystal-induced arthritis (*n *= 4). Unfractionated or NK-depleted synovial fluid lymphocytes (SFL) were purified as described above and this population was, on average, less than 5% CD56^+^.

This study was approved by the ethics committee for clinical research at Hospital Universitario de La Princesa.

### Cell-cell contact assays

PBL or different lymphocyte subsets were incubated for 24 hours in the presence of medium alone or with IL-15 (1 to 100 ng/ml). After being washed, the cells were resuspended in medium and added to 24-well plates (Costar). Unless otherwise stated, then THP-1 cells were added in the proportion 10 lymphocytes to 1 THP-1. As a negative control, lymphocyte–THP-1 cell contact was prevented by using a 0.4 μm pore-size transwell insert (Costar). In some experiments, lymphocytes or THP-1 cells were fixed before cell co-culture (0.05% glutaraldehyde at 4°C for 30 to 45 seconds). After 24 hours the supernatants were harvested and stored at -80°C until the cytokines were quantified.

To investigate the involvement of different cell surface molecules in these experiments, the following purified mAbs were added: MAB002 (negative control), MAB647 (anti-IL15), BAB281 (anti-NKp46), MA152 (anti-NKp80), MAB139 (anti-NKG2D), z199 (anti-CD94/NKG2A), Lia3/2 (anti-CD18), 156-4H9 (anti-CD48) and 2-69 (anti-CD244). All mAbs were used at a final concentration of 10 to 20 μg/ml.

### Induction of IL-15 expression on THP-1 cells

THP-1 cells were stimulated with different concentrations of IFN-γ (1 to 100 ng/ml), TNF (1 to 100 ng/ml), IL-1 (1 to 100 ng/ml) or medium alone for 24 hours; the expression of membrane-bound IL-15 was then analysed by flow cytometry.

### Flow cytometry analysis

Cells were incubated with the specific mAbs at 4°C for 30 minutes. After being washed in PBS, the cells were labelled with fluorescein isothiocyanate-tagged goat anti-mouse Ig (Dako, Salstrup, Denmark) for 30 minutes at 4°C. For double staining, cells were additionally incubated for 15 minutes with mouse serum diluted 1:100 (ICN Biomedicals Inc, Aurora, OH, USA); they were washed and then incubated with a phycoerythrin-conjugated anti-CD56 mAb (Becton Dickinson) for 20 minutes. At least 5 × 10^3 ^cells were analysed with a FACScan flow cytometer (Becton Dickinson).

### Quantification of cytokines in cell-free supernatant

Human TNF concentrations in supernatants were determined by an enzyme immunoassay (EIA). In brief, 96-well high-binding EIA plates (Costar) were coated overnight at 4°C with 50 μl of MAB610 (R&D Systems) per well at 8 μg/ml in PBS, pH 7.4. Subsequently, each well was washed twice with 200 μl of wash buffer (0.05% Tween 20 in PBS, pH 7.4) and blocked for 1 hour by adding 200 μl of PBS containing 2% BSA at 37°C. After each step, the wells were washed three times with 200 μl of wash buffer; 50 μl of dilution buffer (0.1% BSA, 0.05% Tween20, 20 mM Trizma base, 150 mM NaCl, pH 7.3) per well plus 50 μl of each sample or standard dilutions for recombinant human TNF (10,000 to 39 pg/ml; R&D Systems) were then added to the respective wells (in duplicate) and incubated at room temperature for 2 hours. Bound TNF was detected by incubation for 1 hour with, in each well, 50 μl of BAF210 (R&D Systems) diluted to 200 ng/ml in dilution buffer at room temperature. After washing, 100 μl streptavidin HRP (Calbiochem, San Diego, CA) diluted 1:5,000 in dilution buffer was added to each well for 20 minutes at room temperature; the reaction was then developed with 100 μl 3,3',5,5' -tetramethylbenzidine (Chemicon International Inc., Temecula, CA, USA) per well. The optical density of each well was determined with a SpectraII microtitre plate reader (Innogenetics Diagnóstica y Terapéutica, Barcelona, Spain) set to 450 nm, with wavelength correction set to 550 nm. Cytokine values were calculated from the standard curve. Samples that generated values higher than the highest standard were diluted (1:1) in dilution buffer and assayed again.

Because TNF production can vary depending on the lymphocyte donor, in the experiments in which cell–cell interactions were blocked with mAbs the results were normalized with the following equation: TNF production = 100 × TNF_mAb _/TNF_medium _.

Human IFN-γ concentrations were measured with an EIA kit from R&D Systems.

### Statistical analysis

Statistical analysis was performed with Stata 9.1 for Windows (StataCorp LP, College Station, TX, USA), by using one-way analysis-of-variance model with Bonferroni multiple-comparison correction for multiple sample experiments and the Mann–Whitney test for experiments with comparison between two groups.

## Results

### Characterization of a model of TNF production in co-cultures of monocytic cells and IL-15-activated peripheral blood lymphocytes

**Figure 1:**
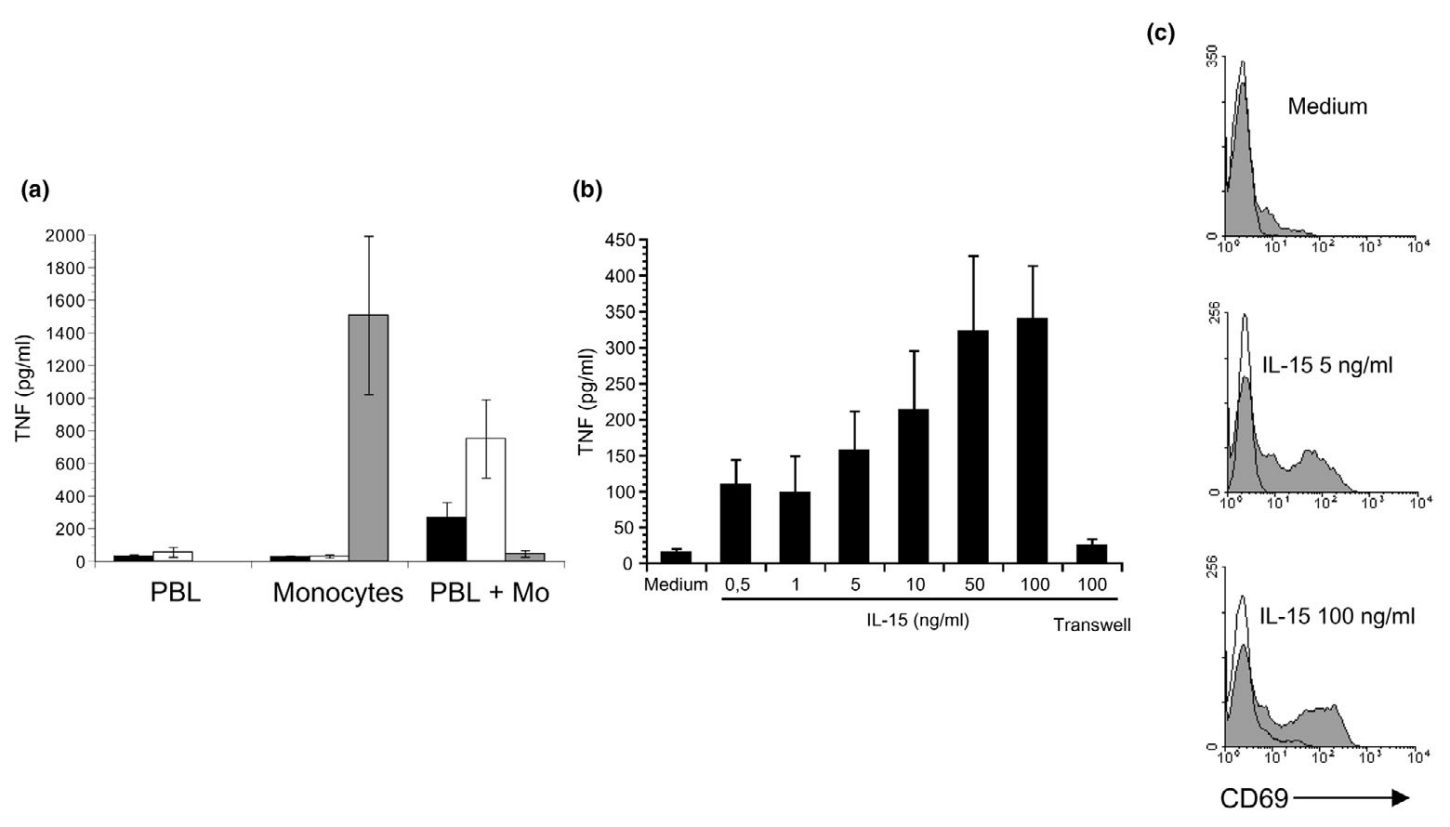
TNF release in co-cultures of IL-15 activated lymphocytes and monocytes. **(a) **Peripheral blood lymphocytes (PBL), monocytes or culture of both cell types were incubated with IL-15 (50 ng/ml; white column) or medium alone (black column) for 24 hours. As a positive control, monocytes were stimulated with lipopolysaccharide (50 ng/ml; grey column in monocytes condition). To determine the effect of intercellular contacts between lymphocytes and monocytes in the presence of IL-15 (50 ng/ml), cells were separated by a 0.4 μm pore transwell (grey column in PBL + Mo condition). TNF was measured in the cell-free supernatants with the use of an enzyme immunoassay. The data are shown as means ± SEM from five independent experiments. **(b) **PBL were stimulated with different doses of IL-15 (0.5 to 100 ng/ml) for 24 hours, and the cells were then washed and co-cultured with autologous monocytes at a 10:1 ratio of PBL to monocytes for a further 24 hours. As a control, the cells in culture were separated by a 0.4 μm pore transwell. TNF was measured in the cell-free supernatants with the use of an enzyme immunoassay. The data are shown as means ± SEM for eight independent experiments. **(c) **PBL were activated as described for (b) and then CD69 expression was analysed by flow cytometry. A representative experiment is shown. The grey histogram depicts CD69 expression and the black solid-line histogram the negative control.

Different *in vitro *models have been described that have raised the importance of intercellular contacts between activated lymphocytes and monocytic cells in the perpetuation of rheumatoid synovitis [9-11]. We have used a model in which PBL are activated with IL-15, a cytokine with a specific presence in the RA microenvironment compared with other arthropathies [17-19]. Neither PBL nor monocytes incubated separately with IL-15 at 50 ng/ml were able to produce TNF (Figure 1a), whereas lipopolysaccharide-activated monocytes secreted large amounts of TNF (Figure 1a; monocytes, grey column). By contrast, when PBL and monocytes were incubated together in the presence of IL-15, a relevant TNF production was observed (Figure 1a). When cell contact between the two cell types was prevented by a 0.4 μm pore transwell, TNF synthesis dropped markedly (Figure 1a; PBL+Mo, grey column). The production of TNF in this model was dependent on the IL-15 dose (Figure 1b) and was correlated with the intensity of PBL activation measured through CD69 expression on the subpopulation that responded to IL-15 (Figure 1c).

**Figure 2:**
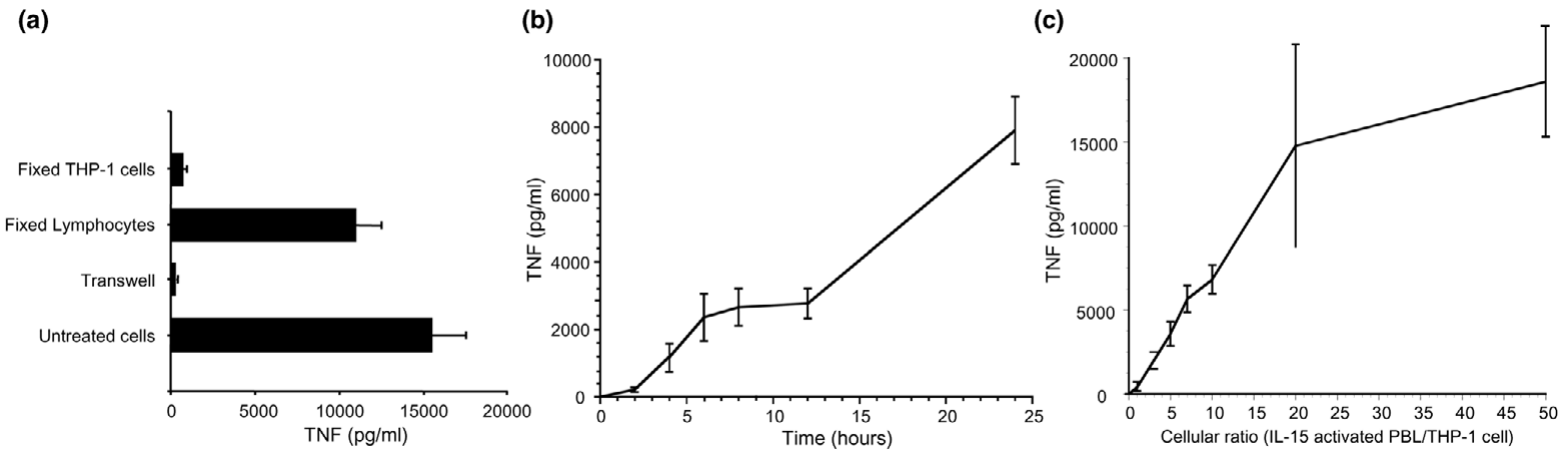
A subpopulation of IL-15-activated PBL induces TNF synthesis in monocytic cells. **(a) **Peripheral blood lymphocytes (PBL) were stimulated with IL-15 at 50 ng/ml for 24 hours and then co-cultured with THP-1 cells for 24 hours; the ratio of lymphocytes to monocytes was 10:1. Under some conditions, lymphocytes or THP-1 cells were fixed with 0.05% glutaraldehyde at 4°C for 30 to 45 s and washed intensively with sterile PBS before co-culture. The data shown are the TNF concentration in the supernatant and are expressed as means ± SEM (*n *= 5). (**b, c) **PBL were stimulated as in (a) and then incubated together with THP-1 cells for different durations (2 to 24 hours) (b) or at different cell ratios (1:1 to 50:1) (c). The data shown are the TNF concentration in the supernatants and are expressed as means ± SEM (*n *= 5).

Similar results were obtained with the monocytic cell line THP-1 (Figure 2a–c). To determine whether TNF secretion was produced by monocytic or lymphocytic cells, experiments were performed with fixed cells. TNF concentration decreased markedly when THP-1 cells were fixed with respect to their basal condition, suggesting that monocytic cells were the main source of this cytokine (Figure 2a). TNF release into the supernatant was dependent on both time (Figure 2b) and the ratio of IL-15-activated PBL to THP-1 cells (Figure 2c). Indeed, TNF release was very inefficient at a ratio of 1:1 and reached a 'plateau' at ratios above 20 activated PBL per THP-1 cell (Figure 2c). These data suggest that the lymphocyte subset that becomes activated by IL-15 and is able to induce TNF production in macrophages seems to be a limiting factor.

### NK cells induce TNF synthesis by monocytic cells

**Figure 3:**
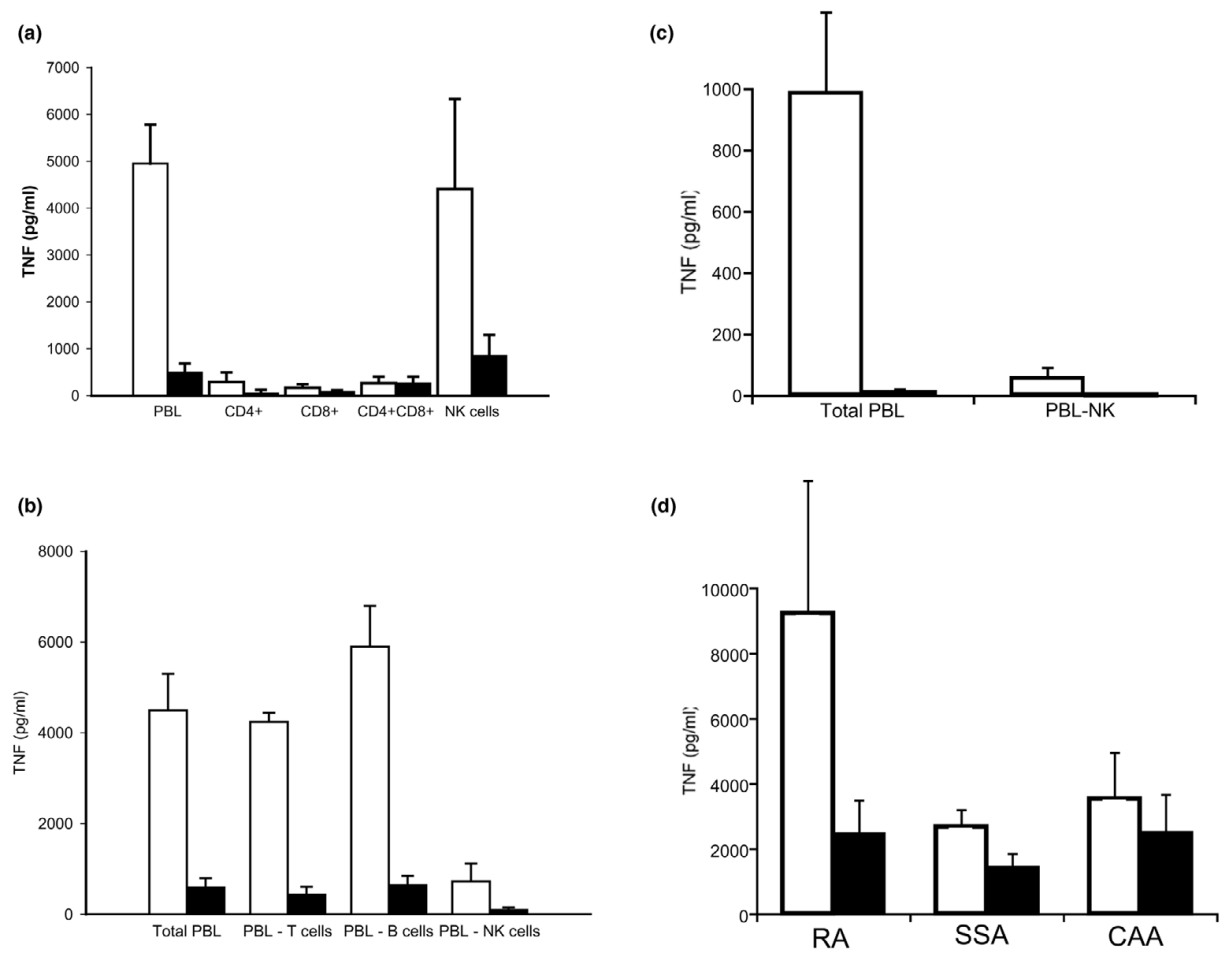
NK cells are the major lymphocyte subpopulation that induce TNF release by monocytes. **(a) **CD4 and CD8 T cells and natural killer (NK) cells were isolated by negative selection from peripheral blood lymphocytes (PBL). Subsequently, purified cells or total PBL were incubated with IL-15 (50 ng/ml; white bars), washed intensively and incubated together with THP-1 cells. To avoid intercellular contact where necessary, cells were separated by a 0.4 μm pore semipermeable membrane (black bars). TNF was measured in cell-free supernatants harvested after 24 hours in co-culture. The data are shown as means ± SEM (*n *= 5). **(b) **PBL were depleted of T cells, B cells or NK cells (PBL – T cells, PBL – B cells and PBL – NK cells, respectively) as described in the Materials and methods section, and the total PBL or the different depleted PBL were then stimulated with 50 ng/ml IL-15 for 24 hours. After being washed, the different PBL groups were brought into contact with THP-1 cells (10:1 ratio of PBL to THP-1 cells; white bars) or the cells were separated in culture by a 0.4 μm pore transwell (black bars) for 24 hours. The data show the TNF concentration in the supernatants and are expressed as means ± SEM from five independent experiments. **(c) **PBL were depleted of NK cells and stimulated as described for (b). After being washed, the different PBL groups were co-cultured with autologous monocytes (white column) at a 10:1 ratio of PBL to monocytes. As in (b), black columns show TNF concentration in supernatants from conditions in which intercellular contacts was prevented by a 0.4 μm pore transwell. The data show the TNF concentration in the supernatants and are expressed as means ± SEM from four independent experiments. **(d) **Synovial fluid lymphocytes (SFL) obtained from knee effusions in different disorders were depleted of NK cells (SFL – NK) as described in the Materials and methods section. Then, both SFL and SFL-NK were allowed to contact with THP-1 cells (10:1 ratio of PBL to THP-1; white bars) or the cells were separated in culture by a 0.4 μm pore transwell (black bars) for 24 hours. The data show the TNF concentration in the supernatants and are expressed as means ± SEM from five samples from rheumatoid arthritis (RA), six samples from seronegative spondyloarthropathies (SSA) and four samples from crystal-associated arthritis (CAA).

Additional experiments showed that the effect of purified NK cells on TNF synthesis by THP-1 cells was similar to that of unfractionated PBL (Figure 3a). In contrast, neither CD4^+ ^nor CD8^+ ^T cells were able to induce any TNF synthesis (Figure 3a). Furthermore, when B cells or T cells were removed from the PBL, their capacity to induce TNF synthesis remained unaffected (Figure 3b). In contrast, the removal of NK cells abrogated this effect almost completely (Figure 3b), a finding that was reproduced when experiments were performed with autologous peripheral blood monocytes (Figure 3c).

Similar results were obtained when we studied the effect of SFL. Hence, when THP-1 cells were incubated with SFL depleted of NK cells, TNF production was significantly lower than that detected in co-cultures of THP-1 with complete SFL (Table 1). To determine whether our findings were specific to RA or were a common phenomenon in most inflammatory arthropathies, we analysed data grouped by different disorders. Interestingly, the highest TNF synthesis was observed in co-cultures of RA SFL with THP-1 cells (Figure 3d). The inhibition of TNF production, when SFL were depleted of NK cells, was therefore stronger in samples from patients with RA (about 75% inhibition) than in synovial fluid from seronegative spondyloarthropathies (50%) or in samples from crystal-induced arthritis (30%; Table 1).

### Monocytic cells induce NK cell activation through membrane-bound IL-15

**Figure 4:**
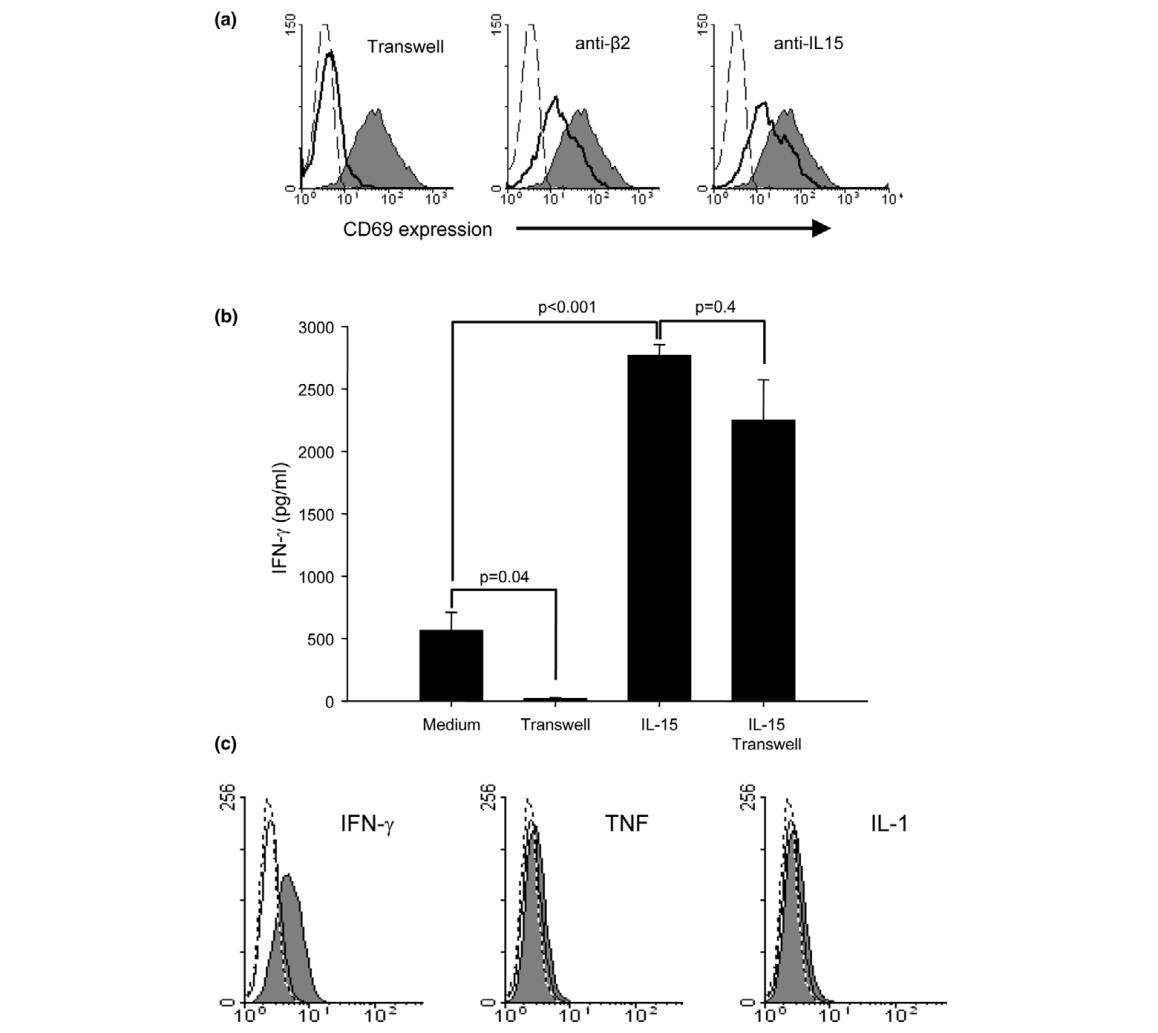
Reciprocal activation between NK and THP-1 cells, the role of IL-15 and IFN-γ. **(a) **IL-15 and β_2 _integrins are involved in the intercellular contact with THP-1 cells that induces the expression of CD69 in natural killer (NK) cells. Peripheral blood lymphocytes (PBL) were co-cultured with THP-1 cells (10:1 ratio of PBL to THP-1) for 24 hours in medium alone or in the presence of an anti-β_2 _integrin mAb (Lia3/2) or an anti-IL-15 mAb (MAB647). As a control, both cell lines were separated by a 0.4 μm pore transwell. A representative experiment of the five performed is shown. The histograms represent the CD69 expression in CD56^+ ^cells in the medium (grey histogram in all panels) or under the different conditions (solid black line in each panel); a negative control is also shown (dotted histogram in all panels). **(b) **Intercellular contact between THP-1 and NK cells induces IFN-γ production. NK cells were cultured in the presence of 50 ng/ml IL-15 for 24 hours, or in medium alone, and the NK cells were then washed and incubated together with THP-1 cells. In some conditions the cells were separated by a 0.4 μm pore transwell and after 24 hours the supernatants were harvested to measure the IFN-γ content with the use of an enzyme immunoassay. The data show the IFN-γ concentrations and are expressed as means ± SEM from six independent experiments. One-way analysis-of-variance model with Bonferroni multiple-comparison correction was used to determine statistical significance. **(c) **IFN-γ increases IL-15 membrane expression in THP-1 cells. Cells were incubated with IFN-γ (100 ng/ml), TNF (100 ng/ml) or IL-1 (100 ng/ml) for 24 hours and then the membrane-bound IL-15 (mIL-15) was measured by indirect immunofluorescence and flow cytometry. A representative experiment of the five performed is shown. Grey histograms represent mIL-15 on stimulated THP-1 cells, solid-line histograms represent basal mIL-15 expression, and the dotted-line histogram is the negative control.

Resting PBL and purified NK cells induce TNF synthesis in monocytes and THP-1 cells to a smaller extent than those previously stimulated with IL-15 (Figure 1a, and data not shown). We therefore assessed the possible effects of THP-1 cells on NK cell activation by measuring the expression of CD69 in these cells. More than 90% of NK cells expressed CD69 on co-culture with this monocytic cell line (Figure 4a and Table 2). This effect was almost totally abrogated when contact between the two cell types was prevented with 0.4 μm transwell inserts or partly prevented by the addition of antibodies against β_2 _integrins or of anti-IL-15 mAbs (Figure 4a and Table 2). Interestingly, this inhibition of CD69 expression was associated with a poorer capacity to induce the synthesis of TNF (Table 2).

In addition, whereas the incubation of resting PB NK cells together with THP-1 cells induced IFN-γ production, this was completely abrogated when both cell lines were separated by a 0.4 μm transwell (Figure 4b). The IFN-γ produced by NK cells prestimulated with IL-15 was significantly higher, but in this case the prevention of intercellular contact with the use of the transwell did not significantly decrease IFN-γ release (Figure 4b).

These findings support the notion that membrane-anchored IL-15 participates in the activation of NK cells after co-culture with THP-1 cells, as has been suggested in previous studies with monocytes and synoviocytes [20,21]. We therefore studied whether the proinflammatory cytokines IFN-γ, IL-1 and TNF modulate IL-15 expression on THP-1 cells, which is very low in resting THP-1 cells. Unlike IFN-γ, neither TNF nor IL-1 was able to induce significant expression of membrane-bound IL-15 in these cells (Figure 4c). Moreover, the effect of IFN-γ was clearly dose-dependent (data not shown).

### The role of NK-cell surface molecules in the induction of TNF synthesis

**Figure 5:**
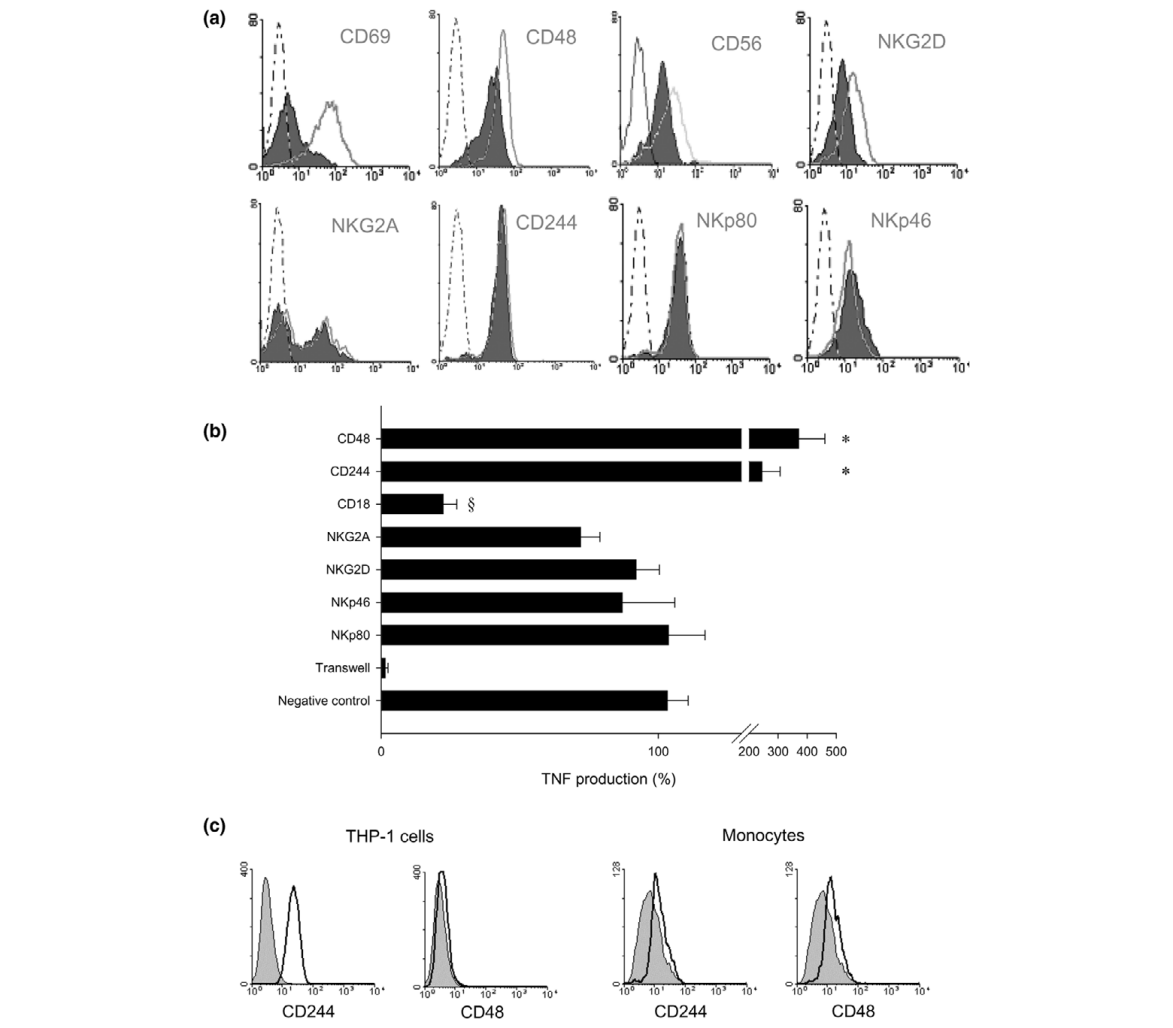
NK cell surface molecules involved in the cell-cell interaction that promotes TNF production by monocytes. **(a) **The effect of IL-15 on the expression of cell surface molecules by natural killer (NK) cells. NK cells were cultured with IL-15 at 50 ng/ml or medium alone for 24 hours, and the expression of different surface molecules was then assessed by flow cytometry. Grey-filled histograms represent the expression of each molecule on resting NK cells, the grey-line histograms represent the expression on IL-15 activated NK cells, and the dotted-line histograms represent the negative control. One representative experiment is shown. **(b) **Effect of different antibodies against NK cell surface molecules on TNF production in co-cultures of NK and THP-1 cells. IL-15-stimulated NK cells were co-cultured with THP-1 cells at a 10:1 cell ratio for 24 hours in the presence of different monoclonal antibodies (see the Materials and methods section for further information). The TNF concentration in cell-free supernatants was quantified with an enzyme immunoassay. The results show the percentage TNF production and are expressed as means ± SEM for eight independent experiments (see the Materials and methods section for definition). Staistical significance:*p < 0.01; ^§^*p *< 0.05; analysis-of-variance test. **(c) **Expression of CD244 and CD48 on monocytes and THP-1 cells. The solid-line histogram represents the expression of each molecule and the grey histogram the negative control.

Exposure to IL-15 increased the expression of CD69, CD56, CD48 and NKG2D by NK cells, although this cytokine did not have any significant effect on other surface molecules such as NKG2A, CD244 (2B4), NKp46 or NKp80 (Figure 5a). To assess the possible influence of these molecules, we performed functional experiments with different mAbs. The mAbs against NKp46, NKp80, NKG2A and NKG2D did not exert any relevant effect on TNF production, whereas the blockage of β_2 _integrins significantly inhibited TNF release (Figure 5b). In contrast, the mAbs against CD244 (2-69 mAb) and CD48 (the CD244 ligand) increased TNF synthesis (Figure 4b). It is noteworthy that NK cells and monocytes express both CD48 and CD244, whereas THP-1 cells express only CD244 (Figure 5a and 5c). Incubation of each cell with anti-CD48 or anti-CD244 mAb did not induce TNF release when these cells were cultured alone (data not shown).

These data suggest that IL-15 enhances the expression of several surface molecules in NK cells. Furthermore, some of these could participate in the intercellular contacts that regulate TNF production by monocytic cells, such as β_2 _integrins, CD48 and CD244.

## Discussion

A significant amount of evidence has accumulated supporting the importance of intercellular contacts in the pathogenic mechanisms underlying RA. Indeed, the importance of these cell-cell interactions in the synthesis and release of pro-inflammatory cytokines and metalloproteinases has been highlighted in several studies [9-11,20,22-24]. Although T cells were thought to be responsible for these activating contacts, our data indicate that NK cells are the main subset of lymphocytes that induce TNF production by monocytic cells in this experimental model of intercellular contact. In fact, considering that NK cells compose about 10% of the PBL, our data suggest that cellular ratios as low as 1 NK cell to 5 or 10 THP-1 cells are able to induce TNF production. Therefore the effects previously assigned to T lymphocytes could indeed be mediated mostly by NK cells. In this regard, although previous works were described to be performed with purified T lymphocytes (more than 90% CD3^+ ^cells), none of them actively employed a strategy to deplete NK cells from their samples [9-11,20,22-24]. Furthermore, here we provide solid evidence that monocytic intercellular contacts with other subsets of PBLs (CD4^+^, CD8^+ ^and B cells) do not induce TNF production.

Our results concur with a recent report describing that activated NK cells induce intracellular TNF expression in monocytes [12]. However, in that work NK cells were purified by positive selection, a procedure that may induce cellular signalling. In contrast, our findings were obtained through negative selection of different subpopulations, avoiding this problem. In contrast, two previous studies described a bidirectional cross-talk between NK cells and dendritic cells leading to mutual activation, but they did not describe the molecules underlying this phenomenon [13,14]. We show here that negatively selected resting NK cells are able to induce TNF synthesis because they are activated by coming into contact with mIL-15 on monocytes. This interaction induces the expression of CD69 on NK cells and also promotes them to synthesize IFN-γ, which in turn upregulates the expression of mIL-15 in resting monocytic cells. Our data therefore support the involvement of monocytes and NK cells in a reciprocal activation loop in which IL-15 and IFN-γ are critical for the sustained production of TNF.

With regard to the specific role of NK cells in different rheumatic conditions, our data show that the capacity to induce TNF release diminished when the SFL were depleted of NK cells. Both effects, namely the induction of TNF synthesis and its inhibition when NK cells were depleted from SFL, were particularly evident in samples from patients with RA. It is conceivable that the activation of macrophages by NK cells, a normal pathway during the initial immune response, might be exacerbated in RA. This might be the consequence of the increased expression of NK-activating cytokines (IL-12, IL-15 and IL-18) in these patients [25]. Indeed, we have already seen that in patients with RA, the serum and synovial fluid levels of IL-15 are higher than in other inflammatory arthropathies [18,19]. Furthermore, a significant correlation between IL-15 serum levels and the expression of mIL-15 on PB monocytes was observed in patients with early arthritis (I Gonzalez-Alvaro, AM Ortiz and Dominguez-Jimenez C, unpublished observation). Accordingly, it would be of interest to determine whether an increased expression of mIL-15 or IL-15 serum levels could identify patients with a more severe disease progression.

We have also generated information about the molecules on activated NK cells that promote TNF production in monocytes. The interaction of CD244 expressed by THP-1 cells with its CD48 ligand on NK cells seems to be involved in regulating the TNF production mediated by intercellular contacts. However, the precise role of these molecules remains to be defined, particularly given that CD244 is known to be an activating receptor in NK cells [26,27]. However, CD244 is also thought to mediate inhibitory responses in the absence of the signalling adaptor protein SAP [28]. Our data may support this inhibitory role because the model renders higher TNF concentrations with THP-1 cells, which lack CD48, than in monocytes that express both CD48 and CD244.

Thus, our findings show that NK cells can engage and stimulate monocytic cells, resulting in the synthesis of TNF. This finding opens the possibility of exploring new therapeutic targets for RA and probably other chronic inflammatory disorders. In this regard, these data further support the application of IL-15 blockage as a treatment for RA, a strategy that has so far provided satisfactory preliminary results [29].

## Conclusion

The main new findings described in this study are as follows. First, NK cells, rather than T lymphocytes, are the main lymphocyte subpopulation involved in the cell-contact-mediated production of TNF that is induced in monocytic cells. This finding may be relevant when considering the pathogenesis of chronic synovitis and it seems to be particularly important with regard to RA. Second, our findings suggest that mIL-15 and IFN-γ contribute to the maintenance of a mutual activator loop between NK cells and monocytes that may result in persistent TNF synthesis. Third, our data also suggest that CD244 and CD48 might regulate TNF production by monocytic cells.

## Abbreviations

BSA = bovine serum albumin; EIA = enzyme immunoassay; FCS = fetal calf serum; IFN = interferon; IL = interleukin; mAb, monoclonal antibody; NK = natural killer; PBL = peripheral blood lymphocytes; PBS = phosphate-buffered saline; RA = rheumatoid arthritis; SFL = synovial fluid lymphocytes; TNF = tumour necrosis factor.

## Competing interests

The authors declare that they have no competing interests.

## Authors' contributions

IG-A participated in the design of the study, performed statistical analysis and drafted the manuscript. CD-J and VN-G purified cells and performed co-culture assays of both PBL and SFL and performed enzyme immunoassays. AMO performed the flow cytometry analysis. PR-N obtained purified NK cells. EF-R and DS participated in the design of the study and helped to draft the manuscript. FS-M participated in the design of the study and its coordination and helped to draft the manuscript. All authors read and approved the final manuscript.

## Figures and Tables

**Table 1:** TNF production in co-cultures of SFL and THP-1 cells: effect of NK cell depletion

Sample source	TNF production (pg/ml)		Inhibition (%)
		
	SFL	SFL – NK	
Rheumatoid arthritis (*n *= 5)	9,237 ± 4,062	2,472 ± 2,472	73.2
Spondyloarthropathies (*n *= 6)	2,680 ± 503	1,407 ± 442	47.5
Crystal-associated arthritis (*n *= 4)	3,557 ± 1,402	2,478 ± 1,196	30.3
Total (*n *= 15)	5,187 ± 1,735	2,059 ± 491	60.3^a^

Where errors are shown, data are means ± SEM. SFL, synovial fluid lymphocytes;

SFL-NK, synovial fluid lymphocytes depleted of natural killer (NK) cells. ^a^Statistical significance: *p *= 0.039, Mann-Whitney test.

**Table 2:** Blockade of CD18 and mIL-15 decreases CD69 expression and TNF production

Substance	Medium	Transwell	anti-CD18	anti-IL15
CD69 (RFI)	69.3 ± 14.9	21.9 ± 13^a^	35.3 ± 10.4^a^	39 ± 12.2^b^
TNF (pg/ml)	9,687 ± 842	559 ± 139^a^	3,594 ± 9,342^b^	5,364 ± 841^b^

The data shown are means ± SEM from eight independent experiments. m-IL-15, membrane-bound IL-15; RFI, relative fluorescence intensity. ^a^Statistical significance: *p *< 0.01 by analysis of variance with Bonferroni multiple-comparison tests. ^b^Statistical significance: *p *< 0.001 by analysis of variance with Bonferroni multiple-comparison tests.
